# Rapid Assembly of Customized TALENs into Multiple Delivery Systems

**DOI:** 10.1371/journal.pone.0080281

**Published:** 2013-11-07

**Authors:** Zhengxing Zhang, Siliang Zhang, Xin Huang, Kyle E. Orwig, Yi Sheng

**Affiliations:** 1 Department of Obstetrics, Gynecology and Reproductive Sciences, University of Pittsburgh School of Medicine, Magee-Womens Research Institute and Foundation, Pittsburgh, Pennsylvania, United States of America; 2 Women’s Cancer Research Center, University of Pittsburgh Cancer Institute, Pittsburgh, Pennsylvania, United States of America; Michigan State University, United States of America

## Abstract

Transcriptional activator-like effector nucleases (TALENs) have become a powerful tool for genome editing. Here we present an efficient TALEN assembly approach in which TALENs are assembled by direct Golden Gate ligation into Gateway^®^ Entry vectors from a repeat variable di-residue (RVD) plasmid array. We constructed TALEN pairs targeted to mouse Ddx3 subfamily genes, and demonstrated that our modified TALEN assembly approach efficiently generates accurate TALEN moieties that effectively introduce mutations into target genes. We generated “user friendly” TALEN Entry vectors containing TALEN expression cassettes with fluorescent reporter genes that can be efficiently transferred via Gateway (LR) recombination into different delivery systems. We demonstrated that the TALEN Entry vectors can be easily transferred to an adenoviral delivery system to expand application to cells that are difficult to transfect. Since TALENs work in pairs, we also generated a TALEN Entry vector set that combines a TALEN pair into one PiggyBac transposon-based destination vector. The approach described here can also be modified for construction of TALE transcriptional activators, repressors or other functional domains.

## Introduction

Generation of genetically modified animal models, as well as creation of mutations and correction of mutants in human cells, have provided many important tools for investigating gene function, genetic disease and drug development [1,2]. Modification of a specific gene/locus in mammalian cells has been a time-consuming task, because it usually involves homologous recombination, which happens at a low rate in nature. However, the efficiency of genetic modification at the target locus could be increased hundreds to thousands fold by creation of a site-specific double-stand break (DSB) through designing “customized” nucleases. Repair of a DSB can occur by two potential pathways: non-homologous end-joining (NHEJ) or homologous recombination (HR). Currently, there are at least four different customized nuclease systems have been developed (see below), and assembly of customized nucleases systems is becoming more efficient [3–6]. 

Zinc-finger nucleases (ZFNs), which emerged a decade ago [7], are artificial nucleases consisting of engineered sequence-specific Cys2His2 zinc-finger DNA-binding domains (3 or more zinc fingers) and a FokI endnucleases cleavage domain. Although tremendous progress has been made to improve the specificity and affinity of zinc finger domains that recognize desired DNA sequences, the target site overlap and crosstalk between individual fingers make it complicated to design and select sequence-specific ZFNs [7], which has impeded researcher access. Homing endonucleases, such as LAGLIDADG family members, have also been engineered for genome editing. Homing endonucleases directly involve DNA sequence recognition and cutting process as well. The drawback is that the production of homing endonucleases targeted to specific sequences appeared to be more complicated [8–11]. 

TALENs, like ZFNs, are also composed of two parts, DNA binding domain and a FokI endnuclease cleavage domain. The DNA binding domain is similar to TALE proteins isolated from plant pathogenic bacteria in *Xanthomonas* genus, whose DNA targeting specificity is determined by tandem 33-35 amino acid repeats, followed by a truncated repeat. The tandem repeats with polymorphisms locate at positions 12 and 13, termed the repeat variable di-residues (RVDs), recognize one of four nucleotides in the target sequence [12,13]. Because TALEs show no context dependency [14], this relationship of RVDs to bases permits engineering of custome TALE RVDs arrays that recognize any defined sequences [15–20] . Initially, some guidelines for TALEN site selection had been suggested [18]. However, subsequent studies suggest that the rules may exist in natural TALE, but are not necessary for designing artificial TALENs, which gives TALEN assembly more flexibility [16,17,19]. A large-scale in vivo analysis reveals that TALENs are significantly more mutagenic than ZFNs [21]. So far, TALENs have been used for genome editing in various organisms, such as different plants [18,22,23], yeast [18,24], fruitflies [25], fish [26–29], hamster [30], mouse [31–33], rat [34] , cattle [35], and human cells [17–20,29,36–40] . TALE transcriptional activators have also been developed to up-regulate gene expression or down regulation by transcriptional repression by replacing the FokI endnuclease cleavage domain with the transcriptional activator VP16 [15,41,42] or transcriptional repressor mSin interaction domain (SID) [43]. 

The CRISPR/Cas system is a defense system evolved in bacteria and archaea that use short RNA to directly degrade foreign nucleic acids. Recently, the *S. pyogenes* type II CRISP/Cas 9 based system has been developed for genome editing in mammalian cells. In this system, the mature crRNA base-paired to trans-activating trans-crRNA that directs the Cas protein to introduce DSBs in target DNA [44–47]. Designing CRISP/Cas 9 system to target a specific sequence is simpler than the TALEN system. However, a relative large-scale analysis demonstrated that it shows significant off-target activity which may limit its application, especially for cell therapy [48]. 

Several different scaffolds and protocols for assembly of TALE RVDs have been reported [15–17,19,20,32]. In this study, based on the TALEN scaffold of Zhang’s group [15], we constructed a set of RVDs and TALEN entry vectors which allow us to efficiently and accurately assemble customized TALENs. Furthermore, through Gateway^®^ LR recombination, the assembled TALEN can be easily transferred into an adenoviral vector system, which facilitates delivery of TALEN into cells. We also demonstrated that a pair of TALENs can be merged into a single plasmid, which is convenient for transfection or injection. Using these modified tools, we generated three pairs of TALENs which target the Ddx3 subfamily of DEAD-box RNA helicase genes in mouse, Ddx3x, Ddx3y and D1Pas1(Pl10) and validated their function by Surveyor mutation analysis and sequencing of target genes. The TALEN assembly system described here has the flexibility for application to other TALE related vectors.

## Materials and Methods

### Plasmid construction

#### A plasmid array encoding TAL repeat variable di-residues (RVDs)

By using the plasmids encoding four RVDs (NI, NN, HD or NG for recognition of A, G, C and T nucleotides, respectively), a total of forty TAL RVDs were amplified by using the primers as reported previously [15]. However, twelve RVDs of the first position of hexamers were re-amplified through primer Ex-F: 5’-GACAGATCT CGTCTC ATGGCCAACCTTAAACCGGCCAACATAC-3’ and reverse primer In-R1: 5’-TCTTATCGGTGCTTCGTTCTCGTCTCCCGTAAGTCCGTGCGCTTGGACAC-3’. Thus, six RVDs can be ligated directly into the intermediate plasmid pTemp-S (see below), rather than the original RVDs, which were ligated to form circular hexamers [15]. All of these RVDs fragments were then cloned into pGEM-T vector (Promega, A3600 ) by TA cloning and sequenced. The resulting RVD plasmid array will be made available for other researchers upon request.

### pTemp-S

A fragment containing LacZ-ɑ was amplified through a forward primer: 5’-gcat cttaagaattcgtctccgaacctaccaagtggtggctatcgagac-3’ and a reverse primer: 5’-cgactctagacgtctccgttcagtaccggtggaaagcgggcagtc-3’ using pFUS_B4 (Addgene #31021) as a template. This fragment was inserted into pFUS_B4 between AflII and XbaI sites to generate an intermediate plasmid, named pTemp-S, to facilitate assembly in subsequent steps. 

### TALEN expression Entry vectors

To generate TALEN Entry plasmids with reporters, we first created plasmid pT2A-EGFP-N3, pEGFP-T2A-C1, pT2A-mCherry-N3 and pmCherry-T2A-C1 by annealing oligos 5’- AATTCTGGCAGTGGAGAGGGCAGAGGAAGTCTGCTAACAT GCGGTGACGTCGAGGAGAATCCTGGCCCAG-3’ and 5’-TCGACTGGGCCAGGATTCTCC TCGACGTCACCGCATGTTAGCA GACTTCCTCTGCCCTCTCCACTGCCAG-3’,which contain the T2A sequence and inserted them into the *EcoR1/SalI* sites of pEGFP-N3 (Clontech, #6080-1) , pEGFP-C1 (Clontech, #6084-1), pmCherry-C1 (Clontech, #632524 ) and pmCherry-N3. We constructed the pmCherry-N3, which is not commercially available, by replacement of the EGFP cDNA between the BamHI and NotI sites in pEGFP-N3 with mCherry cDNA. The primers for mCherry cDNA amplification are forward, 5’- AATGGATCCATCGCCACCATGGTGAGCAAGGGCGAGGAGGAT-3’, and reverse, 5’- GAGTGCGGCCGCTACTTGTACAGCTCGTCCATGCCGCCGGT-3’.

For the standard TALEN Entry plasmids, T2A-EGFP and T2A-mCherry fragments were amplified by PCR with a common forward primer 5’- CGATGAATTCGGCAGTGGAGAGGGCAGA GGAAGTC-3’ and reverse primer 5’-CTCGCTAGCCTA TGGCGCGCCTTACTTGTACAGCTCGTCCA TGCCGAG-3’ for EGFP or reverse primer 5’-CTCGCTAGCCTATGGCGCGCCCTACTTGTACA GCTCGTCCATGCCGCCGGT-3’ for mCherry. The amplified products were inserted into the *EcoRI/XbaI* sites of pEF-ENTR A (Addgene #17427) to create pENTR-EF-T2A-EGFP or pENTR-EF-T2A-mCherry. Using the pTALEN_V2 TALEN plasmids as templates [15], the N-terminal sequences were amplified using forward primer 5’-GATAGATCTACTAGTATGGACTATAAGGACCACGAC-3’ and reverse primer 5’-GGTACATTGAGCAACTGACTG-3’, then digested with *SpeI/NotI*. The C-terminal sequences of the pTALEN_V2 TALEN plasmids were amplified using forward primer 5’- CCAGTTGCTGAAGATCGCGAAGC-3’ and reverse primer 5’- TTAGAATTCTGGCGCGCCTG AGCGGAAATTGATCTCGCCATTG-3’, then digested with *NotI/EcoRI*. These N-teminal and C-terminal fragments were ligated into the *SpeI/EcoRI* sites of pENTR-EF-T2A-EGFP or pENTR-EF-T2A-mCherry (Table 1). 

**Table 1:** TALEN Entry plasmid sets.

**Entry Plasmids^a^**	**Applications**
pENTR-EF1α-TALEN-NN-T2A-EGFP	1. The TALEN Entry plasmid pairs can be used for
pENTR-EF1α-TALEN-NI-T2A-EGFP	TALEN functional tests directly.
pENTR-EF1α-TALEN-NG-T2A-EGFP	2. The TALEN Entry plasmids contain EGFP or
pENTR-EF1α-TALEN-HD-T2A-EGFP	mCherry reporter which can be used to monitor
	transfection efficiency and cell sorting. Both reporters
pENTR-EF1α-TALEN-NN-T2A-mCherry	can be removed from vectors through AscI digestion.
pENTR-EF1α-TALEN-NI-T2A-mCherry	3. T7 RNA polymerase promoter in these vectors
pENTR-EF1α-TALEN-NG-T2A-mCherry	enables in vitro transcription to produce mRNAs.
pENTR-EF1α-TALEN-HD-T2A-mCherry	4. Through LR recombination, the TALEN expression units can be transferred to pAd/PL-DEST to generate TALEN adenoviruses.

^a^Abbreviations: EF1α, elongation factor 1α promoter; T2A,*Thosea asigna* virus 2A peptide

To generate the TALEN ”2 in 1” Entry plasmids, we first removed the EF1α promoter from pEF-ENTR A by HindIII digestion and re-ligation to generate pENTR-Promoterless. This plasmid was amplified by PCR using forward primer 5’-TCCACCATGGGAACCGACATTG-3’ and  reverse primer 5’-GTGTCGACAACTTTTGTATACAAAGTTGGC-3’ and ligated to create pattL5-attL2 (pL5L2). For pattL1-attR5 (pL1R5), the oligos 5’-CAACTTTGTATACAAAAGTTG AACGAGAAACGTAAAATGATATAAATATCAATATATTAAATTAGATTTTGCATAAAAAAC-3’ and 5’-CATAGTGACTGGATATGTTGTGTTTTACAGTATTATGTAGTCTGTTTTTTATGCAAA ATCT-3’ were annealed, then amplified by using forward primer 5’-ACGTTCTA GACAACTTT GTATACAAAAG-3’ and reverse primer 5’-CATCGGATCCATAGTGACTGGATATG-3’. The resulting fragments were inserted into *XbaI/PvuII* sites of pENTR-Promoterless, in which the *attL2* sequence has been removed. The BGH polyA, isolated from pcDNA 3.1, was inserted into *XbaI/XhoI* sites of pL1R5. The EF1α promoter was amplified from pEF-ENTR A using forward primer 5’ ATAAAGCTTGGG AAAGTGATGTCGTGT-3’ and reverse primer 5’-GCTAGCGCTAGCCGACACCTGAAATGGAAGAAA-3’. The chicken β-actin (CAG) promoter was amplified from pCAG-ERT2-CRE-ERT2 (Addgene #13777 ) using forward primer 5’-ATTGATTATTGACTAGTTATTAATAG-3’ and reverse primer 5’-GCTAGCGCTTCTTTGCC AAAATGATGAGAC-3’. Each promoter fragment was digested with *HindIII/NheI* and ligated with EGFP-T2A and mCherry-T2A fragments isolated from pEGFP-T2A-C1 and pmCherry-T2A-C1 via *NheI/KpnI* digestion. The TALEN fragments were amplified from pTALEN_V2 TALEN plasmids using forward primer 5’-GCTAATCGATCTAGATTATGAGCGGAAATTGATCTCGCCATTG-3’

and reverse primer 5’-GGTACCGGTACCATGGACTATAAGGACCACGAC-3’ and digested with KpnI. Then promoters, reporters and TALEN fragments were inserted into XhoI (blunted)/HindIII sites of pL1R5-BGHpA or pL5L2 (Table 2 ).

**Table 2:** Library of TALEN Entry plasmids for 2-in-1 recombination.

**Entry Plasmids** ^a^	**Applications**	
pL1R5-BGHpA-empty		1. The TALEN Entry plasmid pairs can be used for
pL1R5-CAG-EGFP-T2A-TALEN-NN-BGHpA		TALEN functional tests.
pL1R5-CAG-EGFP-T2A-TALEN-NI-BGHpA		2. The TALEN Entry plasmids contain EGFP or
pL1R5-CAG-EGFP-T2A-TALEN-NG-BGHpA		mCherry reporters, which can be used for
pL1R5-CAG-EGFP-T2A-TALEN-HD-BGHpA		monitoring transfection efficiency and cell sorting.
		3. Through LR recombination, the TALEN pairs can
pL5L2-empty		be transferred to pPB-DEST or pPB-Puro-DEST or
pL5L2-EF1α-mCherry-T2A-TALEN-NN		other destination vectors.
pL5L2-EF1α-mCherry-T2A-TALEN-NI		4. TALENs in pL1R5-BGHpA vectors together with
pL5L2-EF1α-mCherry-T2A-TALEN-NG		pL5L2 empty vector can be recombined into
pL5L2-EF1α-mCherry-T2A-TALEN-HD		pAd/PL-DEST to produce adenoviruses with CAG
		EGFP. Alternatively the reverse insertion of
pL5L2-CAG-mCherry-T2A-TALEN-NN		TALENs in pL5L2 vectors together with pL1R5 -
pL5L2-CAG-mCherry-T2A-TALEN-NI		BGHpA empty vector can be recombined into
pL5L2-CAG-mCherry-T2A-TALEN-NG		pAd/PL-DEST to produce adenoviruses with EF1α
pL5L2-CAG-mCherry-T2A-TALEN-HD		mCherry or CAG-mCherry.

^a^Abbreviations: L1 and L2, *attL1*and *attL*2 sites; R5 and L5, *attR5* and *attL5*. CAG, chicken β-actin promoter; T2A,*Thosea asigna* virus 2A peptide; BGHpA, bovine growth hormone polyadenylation sequence; EF1α, elongation factor 1α promoter.

### Destination Plasmids

Adenoviral destination vector pAd/PL-DEST was from Life Technologies (K4940-00). To make *PiggyBac* destination vectors, we replaced the sequence between the SpeI and HpaI sites of pPB-CuO-MCS-IRES-GFP-EF1-CymR-Puro (System Biosciences, PBQM530A-1) with the sequence between the NheI and HpaI sites of pEGFP-N1. The EGFP sequence was then removed by digestion with *BglII/NotI* and replaced with a multiple cloning sites sequence by annealed oligos 5’-GATCCTTAATTAAGACTAGTCGAATT CTGCAGTCGAGGTACCCGGGATCCTCTAGATGC-3’ and 5’-GGCCGCTCTAGAGGATCCCGGGTACCTCGACTGCAGAATTCGACTAGTCTTAATTAAG-3’. Next, the fragment containing *attRI*, chloramphenicol resistant gene, *ccdB* gene and *attLR2* from pWS-TK6 (Addgene #20350 ) was amplified using forward primer 5’-GATCTCTAG ACTCGATTTGTT AGCAGCCTCG-3’ and reverse primer 5’-CGCATTAATTAATCGATGCGATCGCTAGC-3’ and then inserted into *PacI/XbaI* sites of the *PiggyBac* plasmid to generate pPB-DEST. To gain the puromycin selection marker, the fragment containing Pgk-Puro was isolated from pLenti-DEST-Pgk-Puro (Addgene #19068) by *SalI/XbaI* double digestion and inserted into *SalI/XbaI* sites of pPB-DEST. The new plasmid was named pPB-DEST-Puro.

### Assembly of TAL effector RVDs into TALEN expression Entry vectors

We designed TALENs targeted to mouse Ddx3 gene family members, Ddx3x, Ddx3y, and D1Pas1. Ddx3x and Ddx3y are located on the X and Y chromosomes, respectively. D1Pas1 is an intronless retrotransposed copy of the Ddx3x gene and is located on chromosome 1. We designed TALEN pairs to target sequences such as the translation start site or intron/exon junctions that are most likely to disrupt gene expression. In addition, we targeted non-coding sequences, which are distinct among family members. For Ddx3x, the left TALEN target sequences are in the 5’ untranslationed region (5’UTR) of exon 1, and the right TALEN targets 5’UTR close to the coding sequence. For Ddx3y, the left TALEN target sequences are in intron 4, the right TALEN target sequences are in exon 5, and the intervening sequence consists of part of intron 4 and exon 5. For the intronless D1Pas1, the TALEN targets were chosen from the sequences corresponding to exon 1 (left TALEN) and exon 2 (right TALEN) of Ddx3x. This combination is unlikely to mutate Ddx3x or Ddx3y due to the intervening intron sequence. 

For a pair of TALENs, each TALEN targets 20 base pairs of sequences starting from “T” with a space of ~14-20 bases in between. The middle 18 bases of each target sequence (the RVD sequence) was divided into three hexamers that were ligated into pTemp-S [15] . The RVDs were assembled into TALEN Entry basic vectors through two continuous golden gate ligation [49]. The detailed protocol is in Supplementary Methods (Protocol S1 A). Half (~7 µl) of each final ligation products were used to transform chemical competent DH 5α cells. Six colonies were inoculated from each ligation. Plasmid DNAs were extracted and screened by SalI digestion, then sequencing using primer TALE-Seq-F1, TALE-Seq-F2 and TALE-Seq-R1 [15]. 

### Transfer of assembled TALENs to the Destination Vectors

To transfer the assembled TALENs into adenoviral destination vectors, 150 ng of TALEN Entry vector and 150 ng destination vector pAd/PL-DES), 2 µl Gateway^®^ LR Clonase^®^ II Enzyme Mix (Life Technologies, 11791-020) and TE buffer (pH 8.0) were added up to 10 µl. After incubation at room temperature for 2 hours, 4 µl of the product were directly added into 50 µl DH5α competent cells for transformation.

For the “2 in 1” system, two TALEN Entry vectors (150 ng each), the destination vector pPB-DEST or pPB- DEST-Puro (150 ng), 2 µl Gateway^®^ LR Clonase^®^ II Plus Enzyme Mix (Life Technologies, 12538-120) and TE buffer (pH 8.0) were added up to 10 µl . After incubation at room temperature for 4 hours to overnight, 4 µl of the product were directly added into 50 µl DH5α competent cells for transformation. The restriction digestion maps of all plasmids are presented in Protocol S1 B.

### Production of Adenoviral vectors

Adenoviral vectors were produced in 293A cells according to the instructions of ViraPower^TM^ Adenoviral Expression System (Life Technologies, K4940-00). To determine the titers, the viruses were serially diluted and added to 12-well plates pre-seeded with HEK 293 cells. Seventy hours after transduction, EGFP or mCherry expressing cells or colonies of cells were counted to calculate plaque-forming units or transducing units.

### TALEN plasmid transfection and viral vector transduction

A Neuroblast cell line Neuro-2a (ATCC^®^ CCL-131^™^) was used for validation of TALEN function. The day before transfection, 2-3x10^5^ cells were seeded per well in a 24-well plate. TALEN entry vector pairs (0.8 µg for each TALEN) or 1 ug for “2 in 1” vectors, were transfected into cells twice on two consecutive days using Lipofectamine 2000 (Life Technologies). The cells were collected 72 hours after the first transfection and genomic DNA was extracted for further analysis. For TALENs targeting Ddx3y gene (on Y chromosome), primary fibroblasts derived from C57BL/6 male mouse tail tips were used. All animal procedures were approved by the Institutional Animal Care and Use Committee (IACUC) of the University of Pittsburgh, which is the IACUC of record for Magee-Womens Research Institute (Assurance number A3187-01), in accordance with the National Institutes of Health Guidelines for the Care and Use of Laboratory Animals. Mouse G4 embryonic stem cells and rat C6 glial tumor cells (ATCC^®^-CCL-107^™^) were cultured with standard protocols. Different cell lines were transduced with adenoviral vectors using a multiplicity of infection (MOI) of 2-5. 

### Surveyor^®^ mutation analysis and sequencing

Genomic DNA in plasmid transfected or virus transduced cells was extracted and the genomic regions encompassing the TALEN target sites in mouse Ddx3x, Ddx3y and D1Pas1 were amplified by PCR. The primers for amplification of Ddx3x were: forward 5’-aagatt aggggaccggtgg- 3’, reverse 5’-gacgggaaggaaaaagca- 3’; for Ddx3y: forward 5’-gcccagcagtttgagctatt-3’, reverse 5’-gccatccttctgaccctgta-3’; and for D1Pas1: forward 5’-gccatagcgttagcttggag-3’, reverse 5’-catcctcgtctgctttgtca-3’. Genomic mutation assay was performed according to SURVEYOR Mutation Detection Kit (Transgenomic, 706020). Each experiment was replicated a minimum of three times to establish the reproducibility of the method. The PCR products with mutations were subcloned into Zero Blunt PCR kit (Life Technologies, K2700-20) and sequenced. 

## Results

### TAL effector RVD plasmid array

To generate TALEN constructs targeted to genes in mammalian cells, we adopted the TALEN toolbox from Zhang’s group [15] with modifications described here. The assembly of TALENs by PCR in the original strategy is straightforward, but we frequently observed mutations or deletions that had been introduced into TALENs, probably because tandem repeats of the RVD sequences caused PCR errors. Therefore, we cloned the TAL effector RVD in into pGEM-T vector to generate a RVD array of forty plasmids for hexamer construction, and one intermediate plasmid, pTemp-S. For TALEN assembly, while position 2 to 5 of all hexamers share the same plasmids encoding RVDs, NI (A), HD (C), NG (T), or NN (G) respectively, different plasmids should be picked for the first and last RVDs for different hexamers (Figure S1, Top ) to facilitate ligation of RVD sequences to generate each hexamer. The same intermediate plasmid pTemp-S is used for all hexamer assembly. Generally, it is not necessary to transform hexamer ligation products and screen the plasmids. However, this is an option if the TALEN assembly by one-step transformation fails (Figure S1, Bottom).

### TALEN Entry vectors

The Gateway^®^ technology (Life technologies), which is based on the bacteriophage lambda site-specific recombination system, is very efficient and specific for molecular cloning. In this system, the sequences in the first plasmid (Entry vectors) can be transferred to the second plasmid (Destination vector) through *attL/attR* (LR) recombination [50,51], which is suitable for choosing different TALEN delivery systems in the future. 

We generated two sets of four TALEN Entry vectors containing either EGFP or mCherry reporters, which are driven by human EF1α promoter (Table 1). T7 RNA polymerase promoter following EF1α promoter in these vectors can be used for in vitro transcription to produce mRNAs. 

**Figure 1:**
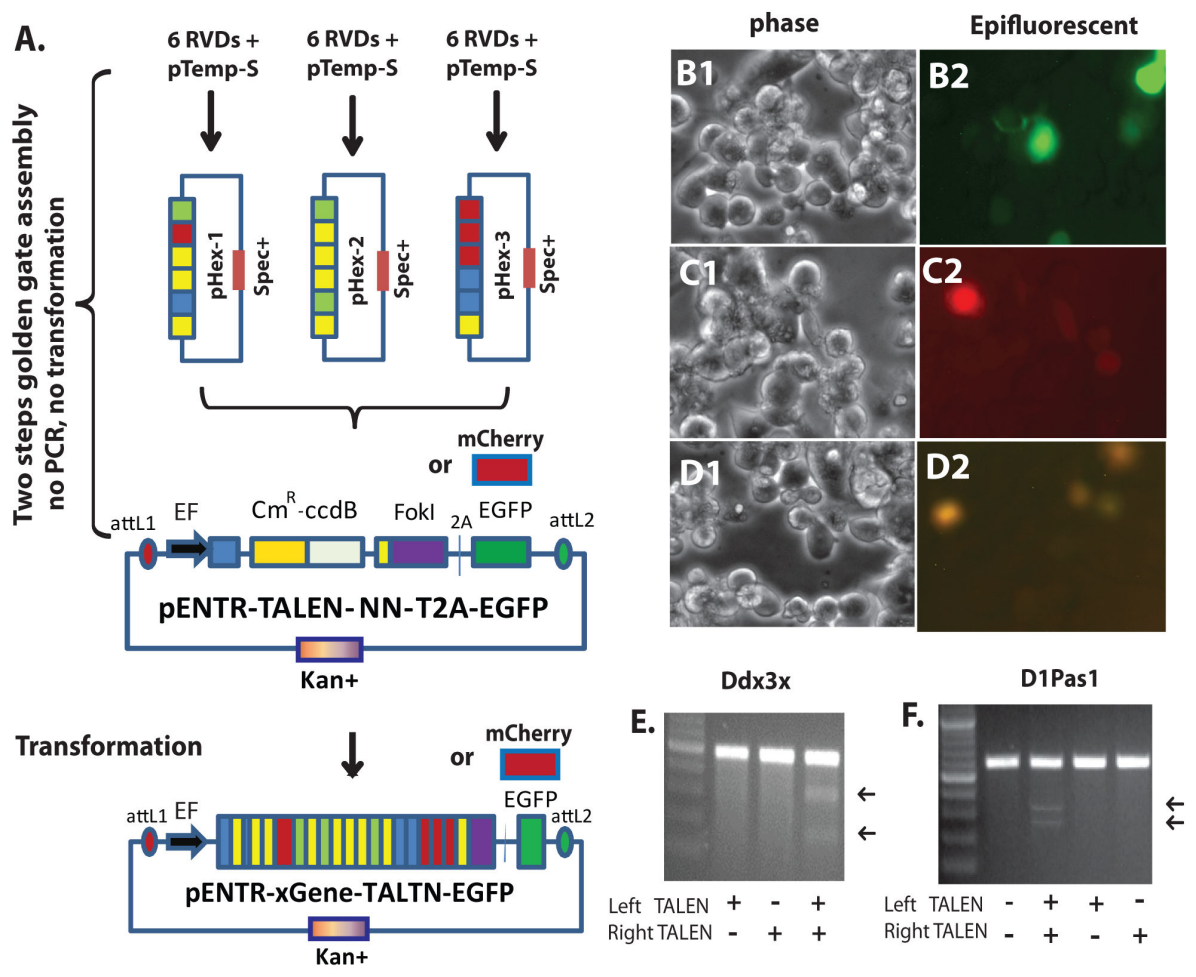
Assembly of RVDs into TALEN Entry plasmids and validation of TALEN function. A. Repeat variable di-residues (RVDs, see Figure S1) are assembled by golden gate ligation into pTemp-S intermediate plasmids (pHex1-3), which are subsequently combined by golden gate ligation into the pENTR-TALEN entry vectors. No PCR amplification is required. TALEN entry vectors contain mCherry or EGFP reporter genes. B-D. Example of transfection of Ddx3x TALEN Entry plasmids into mouse Neuro-2a cells. B1-B2, Ddx3x left TALEN (with EGFP reporter); C1-C2; Ddx3x right TALEN (with mCherry reporter); D1-D2, Ddx3x TALEN pair. B1, C1, D1, Phase contrast; B2, C2, D2, epifluorescence. E. Surveyor mutation analysis of Ddx3x gene after transfection of Ddx3x TALENs vectors into Neuro-2a cells. F. Surveyor^®^ mutation analysis of D1Pas1 gene after transfection of D1Pas1 TALENs vectors into Neuro-2a cells. RVD, repeat variable di-residues. EF, human EF1α promoter. Cm^R^, Chloramphenicol resistance gene. ccdB, a lethal gene targeting DNA gyrase. FoKI, FoKI C-terminal domain. 2A, *Thosea asigna* virus 2A peptide. Spec^+^, Spectinomycin resistance gene.

We then used the new TALE RVD plasmid array and TALEN Entry vectors to construct TALEN pairs to target mouse Ddx3 gene family members (Ddx3x, Ddx3y and D1Pas1). Each TALEN binding sequence and RVDs are shown on Figure S2. Using a two continuous golden gate ligation approach (Figure 1A, Protocol S1), we routinely obtained 4 to 6 out of 6 randomly picked colonies with the correct sequences. Sequence analyses did not reveal any mutations if the plasmid had the correct restriction enzyme patterns. Except of those RVDs which were synthesized chemically and ligated into TALEN expression vectors [17,19,34], the comparison of our strategy to others is provided in Table S1. We transfected TALEN Entry plasmids targeted to Ddx3x and D1Pas1 into mouse Neuro 2a cells. The transfection efficiency and TALEN expression were easily monitored via expression of fluorescent reporter genes, which were encoded from the same transcript as the TALENs with intervening 2A sequences (Figure 1B-D). We favored this approach over the approach of co-transfecting with a separate reporter plasmid [26,29]. Furthermore, if the donor DNAs containing either green or red fluorescent reporter are used for homologous recombination, EGFP or mCherry in these customized TALEN vectors can be simply removed by AscI digestion and re-ligation. Surveyor^®^ mutation analysis was performed to validate the activity of each TALEN pair. As shown in Figure 1E and F, any single TALEN (left or right) vector targeting Ddx3x or D1Pas1 did not introduced mutations around the target sequences. However, Co-transfection of TALEN pairs resulted in mutagenesis that was revealed by Surveyor mutation analysis. 

### Transfer of customized TALENs to adenoviral vector

**Figure 2:**
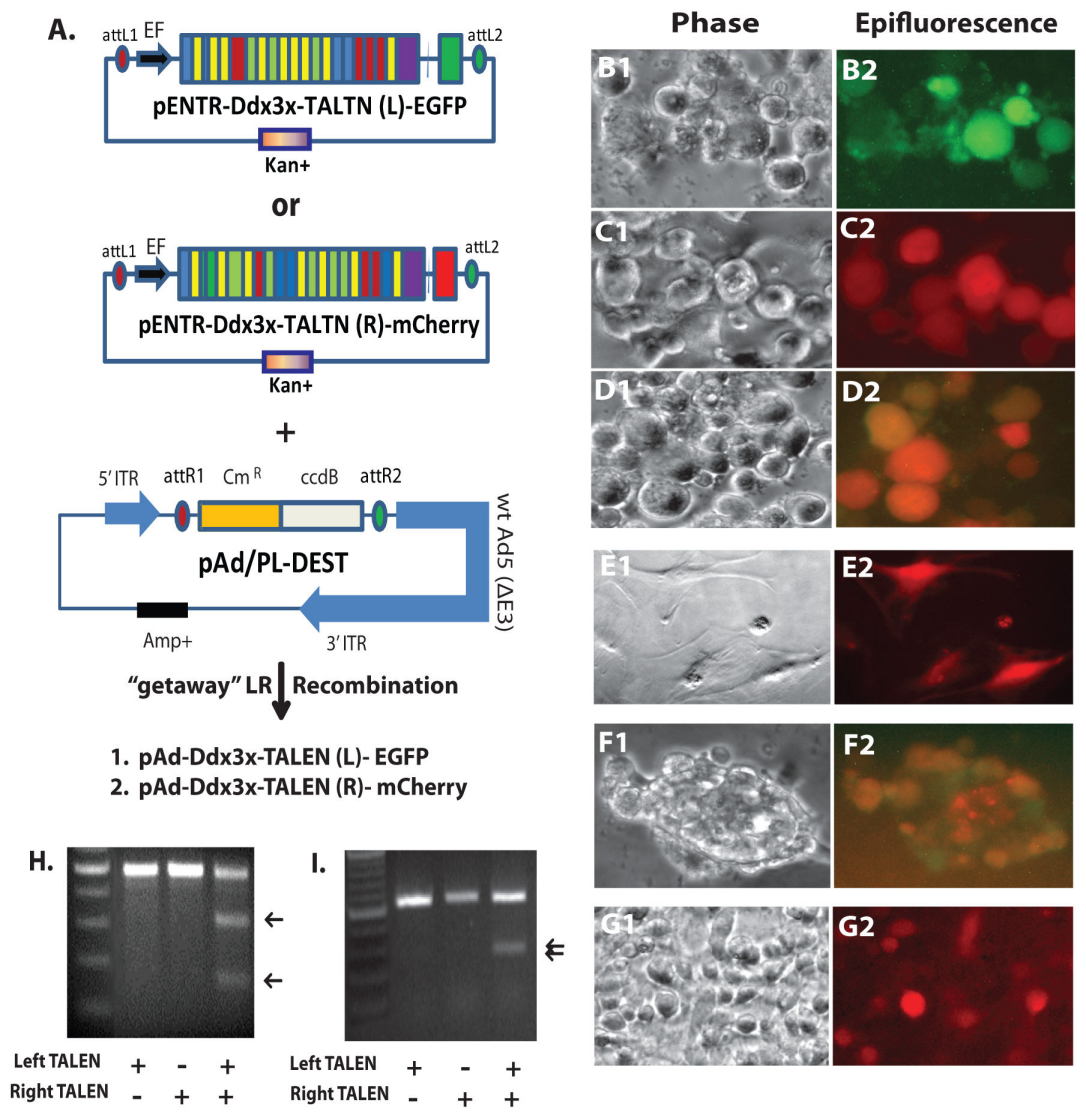
Transfer of TALEN sequences into adenoviral vectors and validation of TALEN function. A. The left and right TALEN sequences described in Figure 1 were transferred into an adenoviral destination vector, pAd/PL-DEST, by Gateway® LR recombination. B-D, Example of transduction of Ddx3x TALEN adenoviruses into mouse Neuro-2A cells. B1-B2, left Ddx3x adenoviral-TALEN. C1-C2, right Ddx3x adenoviral-TALEN. D1-D2, Ddx3x adenoviral TALEN pair. E1-E2, Transduction of Ddx3y adenoviral-TALEN pair (both linked to mCherry) into male mouse fibroblasts. F1-F2, Transduction of Ddx3x adenoviral TALEN pair into mouse ES cells; left TALEN linked to EGFP and right TALEN linked to mCherry. G1-G2, Transduction of Ddx3y adenoviral-TALEN pair (both linked to mCherry) into rat C6 cells. H. Surveyor^®^ mutation analysis of Ddx3x gene after transduction with Ddx3x adenoviral-TALENs into Neuro-2a cells. I. Surveyor^®^ mutation analysis of Ddx3y gene after transduction of Ddx3y adenoviral-TALENs into male mouse fibroblasts.

Transfer of TALENs to non-integrating adenoviral vectors would theoretically significantly enhance the efficiency of TALEN delivery and broaden the potential applications of TALEN technology. Therefore, we recombined TALEN pairs, which targeted either Ddx3x or Ddx3y respectively, from their Entry vectors into adenoviral destination vectors pAD/PL-DEST (Figure 2A). The TALEN Entry vectors can be easily transferred to adenoviral vectors by Gateway^®^ LR recombination.

We then generated TALEN adenoviruses for Ddx3x and Ddx3y and tested them in either Neuro-2a cells or mouse fibroblasts. Based on reporter gene expression shown in Figure 2B-D, the efficiency of Ddx3x TALEN adenovirus transduction into Neuro-2a cells was qualitatively greater than that of sequential (2x) TALEN plasmid transfections (Compare Figure 1B-D and Figure 2B-D). Surveyor^®^ mutation analysis demonstrated that adenovirus transduction with single TALENs did not cause mutations at their target region, while adenovirus transduction with TALEN pairs did (Figure 2H). The ratio of cut to non-cut DNA from adenovirus-TALEN transduced samples was approximately 50% (Figure 2H). Thus, higher efficiency of adenoviral vector transduction resulted in higher genomic mutagenesis (compare Figure 2H and Figure 1E). Similar results were obtained for Ddx3y TALEN adenovirus pairs, which were tested in mouse male fibroblasts (Figure 2F and I). To show the tropism of virus and ubiquity of human EF1α promoter, the adenoviruses were also transduced into mouse G4 ES cells and rat C6 glioma cells (Figure 2E and G). During the preparation of this manuscript, we note that Holkers and coworkers used a different plasmid system to introduce TALEN sequences in adenoviral vectors [52]. However, their cloning strategy required several extra steps (see Discussion).

### Sequence analysis of Ddx3 genes after treatment with plasmid or adenoviral TALENs

**Figure 3:**
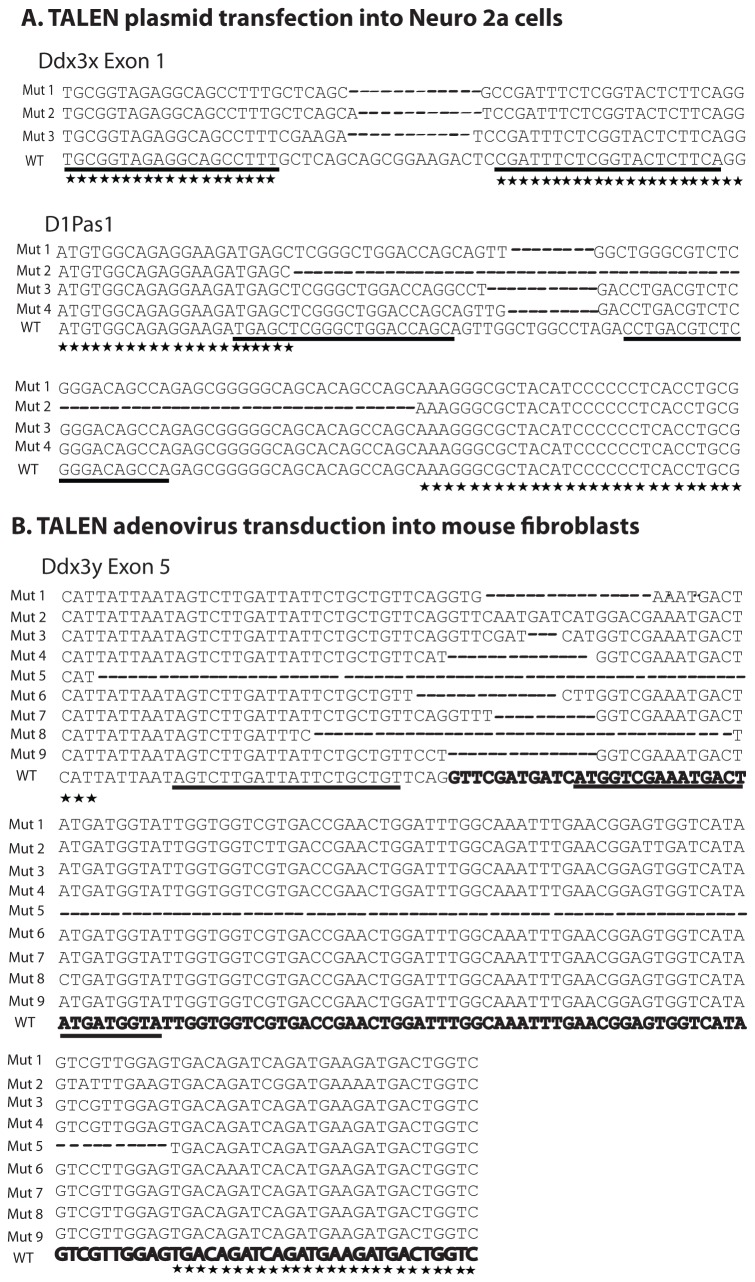
TALEN-induced mutations in mouse Ddx3 subfamily genes. A. DNA sequence analysis of Ddx3x and D1Pas1 genes in Neuro 2A cells after transfection with plasmid TALENpairs. B. DNA sequence analysis of Ddx3y gene in male mouse fibroblasts after transduction with adenoviral-TALEN pairs. TALEN binding sequences are underlined. Stars indicate the identical sequences. Dashes indicate missing nucleotides compared to the wild type (WT) sequences. Exon 5 sequences of Ddx3y are in bold.

We cloned the PCR products amplified at the target regions of Ddx3x and D1Pas1 from Neuro-2a cells which were transfected with TALEN plasmid pairs (Figure 3A), and Ddx3y from male mouse fibroblasts transduced with adenoviral TALEN pairs (Figure 3B). We confirmed that the cells treated with customized TALENs generated mutations around their target sequences (Figure 3). The largest deletion detected in Ddx3y was 193 bp (not shown).

#### Assembly of customized TALEN pair into one vector-“2 in 1”

Because of the feature of FokI, TALENs must work as a pair. We believe it should be beneficial for plasmid transfection and DNA injection to engineer a customized TALEN pair into one plasmid. We thought it may be feasible to clone both left and right TALENs into one destination vector via the Multisite Gateway^®^ Pro system [53] (Life technologies). To enable this approach, different *att* sites must be chosen for recombination of two or more fragments into the Entry vectors.

**Figure 4:**
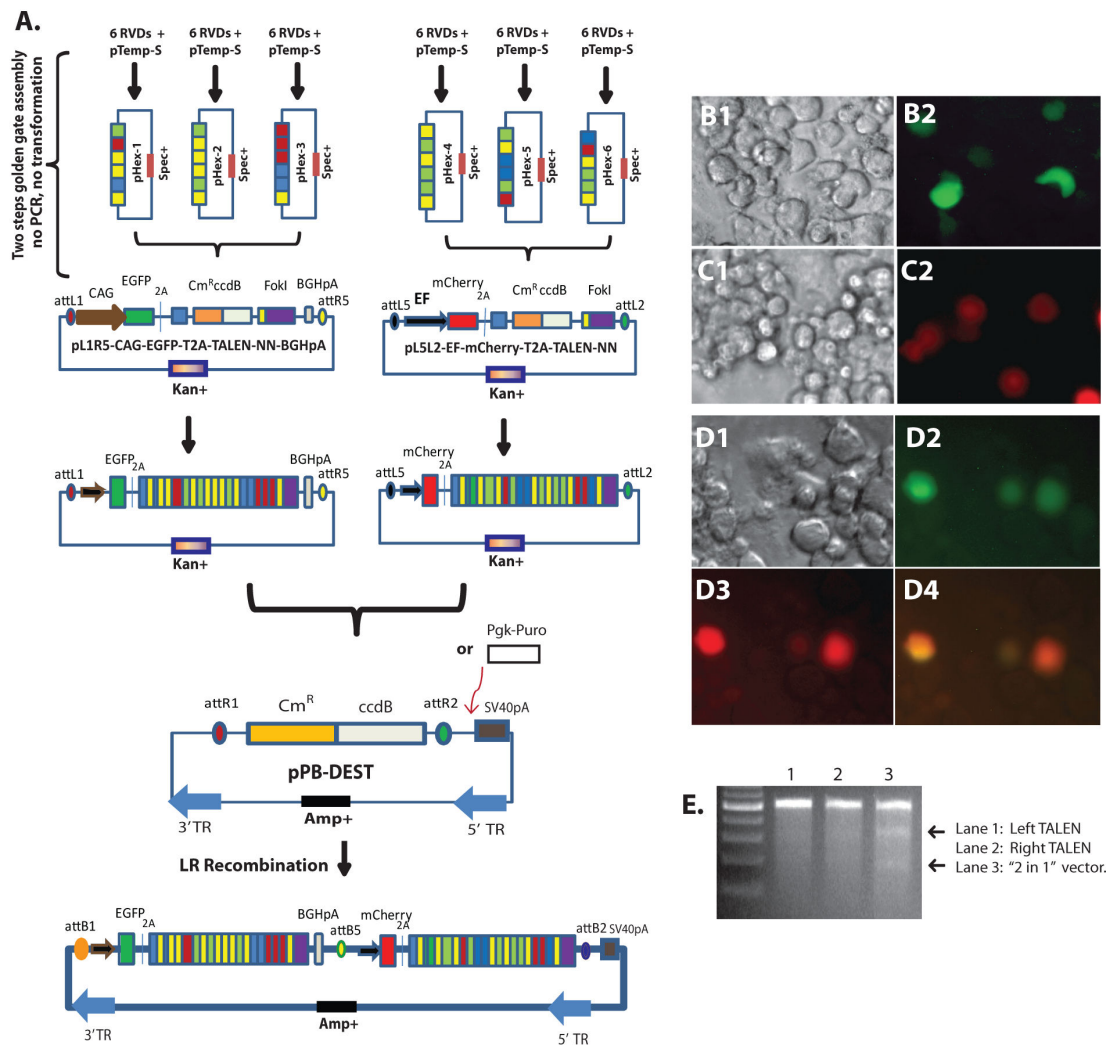
Combination of a TALEN pair into one plasmid vector. A. Left and right TALENs were ligated into different Entry plasmids to produce the “2 in 1” TALEN system. The left TALEN Entry vector contained *attL1* and *attR5* flanking sequences and the EGFP reporter. The right TALEN Entry vector contained attL5 and attL2 flanking sequences and the mCherry reporter. The left and right TALEN Entry vectors were then combined into the pPB-DEST destination vector by Gateway® LR recombination. In some cases Pgk-Puro was added to the destination vector to facilitate selection. B-D Example of transfection of Ddx3x TALEN plasmids into mouse Neuro-2a cells. B1 and B2: left Ddx3x TALEN Entry plasmid. C1 and C2, right Ddx3x TALEN Entry plasmid. D1 to D4, “2 in 1” Ddx3x TALEN plasmid. B1, C1, D1, phase contrast; B2, C2, D2, D3, D4, epifluorescence. E. Surveyor mutation analysis of the Ddx3x gene after transfection of the individual left (lane 1) or right (lane 2) Ddx3x TALEN Entry plasmids or the “2 in 1“ Ddx3x TALEN plasmid (lane 3) into Neuro-2a cells.

We generated 12 TALEN Entry constructs. Four vectors, which have *attL1* and *attR5* flanking sequences and EGFP reporter for one side TALEN (e.g.,left TALEN) assembly, and the other eight vectors, which hold *attL5* and *attL2* flanking sequences and mCherry reporter for the other side TALEN (e.g., right TALEN) assembly (Table 2). The assembly of RVDs into these Entry plasmids followed the same procedure described above. We also constructed two PiggyBac transposon-based destination vectors, pPB-DEST and pPB-DEST-Puro (Figure 4A). This system can be readily modified for other destination vector systems. 

As a proof of concept, we generated a pair of Entry TALEN vectors targeting exon 1 of the Ddx3x gene using same RVDs described above. We then recombined into the pPB-DEST and pPB-DEST- Puro destination vectors. The two fragment recombination was effective, despite the fact that thirty-six RVDs (eighteen RVDs for each side TALEN) were present in the sequences. It should be pointed out that it is impossible to sequence the RVDs in the final vectors. Nonetheless, we transfected each TALEN Entry plasmid (Figure 4B and C) and the TALEN “2 in 1” plasmid (Figure 4D) into Neuro-2a cells. Surveyor^®^ mutation analysis demonstrated that the TALEN pair assembled in one plasmid (“2 in 1”) was capable of introducing mutations in genome (Figure 4E). 

## Discussion

Several groups have reported different systems for customized TALEN assembly [15–17,19,20,32,36,38,54–56]. Most reports have utilized plasmid-based TALEN expression systems and tested function by transfection of TALEN pairs into cell lines which were easily transfected by lipofection [15,17–19,29,57,58], electroporation [16,31,59,60] or direct injection of DNA or mRNAs [21,32,59–61]. However, many cell lines and primary cells are difficult to transfect efficiently. In many cases, this obstacle can be circumvented using viral vectors. These challenges prompted us to devise methods to facilitate accurate TALEN assembly (RVD plasmid array and Entry vector) and design efficient delivery systems (adenovirus and 2 in 1 plasmids). 

Our TALEN assembly approach exploits the features of the methodology originally described by Sanjana and colleagues [15]. However, we assembled TALENs using a sequence validated array of RVD plasmid encoding repeat monomers, which eliminated possible errors introduced by PCR. We used these basic materials to assemble TALENs targeting three genes of Ddx3 gene family, which are located on chromosome 1 (D1Pas1), the X chromosome (Ddx3x) and the Y chromosome (Ddx3y) and did not observe any mutations in our assembled TALEN constructs. The system described here saves the time and labor of amplification and purification of PCR products, relative to PCR only [15,20,24,54] or PCR-plasmid mixed assembly [16] approaches. In addition, our approach eliminated an extra step of transformation compared to other plasmid-based assembly approaches [18,29,38,62]. 

The TALEN Entry vectors generated in this study (Table 1) contain fluorescent reporters (EGFP or mCherry), which help to evaluate the transfection efficiency and can facilitate tracking and sorting of cells containing the TALEN moiety. Furthermore, the new TALEN Entry vector sets are in a Gateway^®^ Entry vector-based system, which can be easily transferred to other delivery system, such as viral or transposon vectors through in vitro LR recombination. Indeed, we successfully transferred TALEN-reporter expression units into adenoviral plasmids without losing any TALEN expression unit sequences. Adenoviral vectors efficiently transduce dividing or non-dividing mammalian cells and can be produced at high titers. We demonstrated in the current study that adenoviral TALENs can effectively modify target gene sequences. During preparation of this manuscript, Holkers and co-workers reported similar success using adenoviruses to introduce TALEN sequences into HeLa cells with a different strategy [52]. To generate adenoviral TALEN plasmid in that study, the authors first assembled the TALEN sequence into an expression vector, then isolated the TALEN sequences and inserted them into a shuttle vector, followed by HR in *Escherichia coli* BJ5183^pAdEasy-1^ to get adenoviral TALEN vectors [52]. In the current study, we report a relatively simple strategy in which adenoviral TALEN vectors are assembled directly from TALEN Entry plasmids in the test tube via LR recombination. The fact that adenoviral vectors rarely integrate in the genome may be advantageous in some experimental circumstances. For some studies, the adenovirus-TALEN approach described here may provide a viable alternative to lentiviral vector-based small RNA knockdown systems that are currently widely used. 

We also built a set of TALEN Entry plasmids with different *att* flanking sequences, which can be used to join TALEN pairs into PiggyBac transposon-based destination vectors (2 in 1 system). The targeting efficiency of one plasmid transfection should theoretically be higher than that of two plasmids. The individual Entry TALEN vectors for the “2 in 1” system can also be recombined to pAd/PL-DEST adenoviral vectors. However, because of adenovirus genome size limitations, the new generation of gutless adenoviral vectors [63] may be required to assemble the “2 in 1” TALEN system in adenoviruses. Cloning of TALEN expression units into PiggyBac vectors could also increase transfection efficiency if co-transfected with transposase. This system may not be limited for TALEN assembly. For example, by replacement of the FokI C-terminal domain in TALEN vectors with transcriptional activator or repressor domains, and generation of new multisite Gateway^®^ Entry plasmids, we could regulate 2 to 4 genes by using a single plasmid because the PiggyBac transposon system has been shown to deliver cargo sizes larger than 200 kb [64–67]. Furthermore, integrated TALE PiggyBac transposons can be removed from the genome by transient expression of transposase without leaving a “footprint” [68,69].

Future advances in the TALEN field will improve specificity, which is important for cell therapy. This could be done, for example, by adding new Asn-His (NH) or Asn-Lys (NK) RVDs, which have high biological activity and specificity for G [43,57] into the array or by use of mutated FokI C-terminal domain DD and RR in TALEN Entry vectors to reduce off-target nuclease activity [68]. In this work, we developed a robust and accurate TALEN assembly system and “user friendly” TALEN Entry vectors that can be modified by basic molecular biology methods to take advantage of new developments in the TALEN field. 

## Supporting Information

Figure S1 **Repeat variable di-residue (RVD) plasmid library and hexamer assembly.** Upper: RVDs library plasmids for TALEN assembly. Lower: example of assembly of 3 hexamers for the left TALEN of exon 1 of Ddx3x.
(PDF)

Figure S2 **TALENs’ target sequences of Ddx3x, Ddx3y and D1Pas1.** The TALEN pair for Ddx3x targets the 5’ untranslated region. The TALEN pair for Ddx3y targets intron 4 (left TALEN, red) and exon 5 (Right TALEN, blue). The TALEN pair of the D1Pas1 (retrotransposed autosomal copy of Ddx3x) targets sequences corresponding to exon 1 (for left TALEN, red) and exon 2 (for right TALEN, blue) of Ddx3x. The left and right TALEN target sequences for each gene are indicated with bold underlines. The assembled RVDs (with encoded nucleotides) for the left and right TALENs are below each target gene region. RVDs (NI, NG, NN and HD) are color coded in correspondence with the RVD library in Figure S2. Exon sequences are indicated with bold font.
(PDF)

Protocol S1 **Protocol.**
(PDF)

Table S1 **Comparison of the Strategy of TALEN Assembly.**
(PDF)
