# Correction: Rapid Assembly of Customized TALENs into Multiple Delivery Systems

**DOI:** 10.1371/annotation/25d8fb69-9279-4eaf-a1c8-675aa73f7e2d

**Published:** 2014-01-02

**Authors:** Zhenxing Zhang, Siliang Zhang, Xin Huang, Kyle E. Orwig, Yi Sheng

The name of the first author was spelled incorrectly. The correct name is: Zhenxing Zhang.

