# Supplementary material for: Rapid Assembly of Customized TALENs into Multiple Delivery Systems
Source: PLoS One. 2013 Nov 7;8(11):e80281. doi: 10.1371/journal.pone.0080281 (PMC3820630; doi:10.1371/journal.pone.0080281)
Supplement: Figure S1 — Repeat variable di-residue (RVD) plasmid library and hexamer assembly. Upper: RVDs library plasmids for TALEN assembly. Lower: example of assembly of 3 hexamers for the left TALEN of exon 1 of Ddx3x. (PDF) [file pone.0080281.s001.pdf]

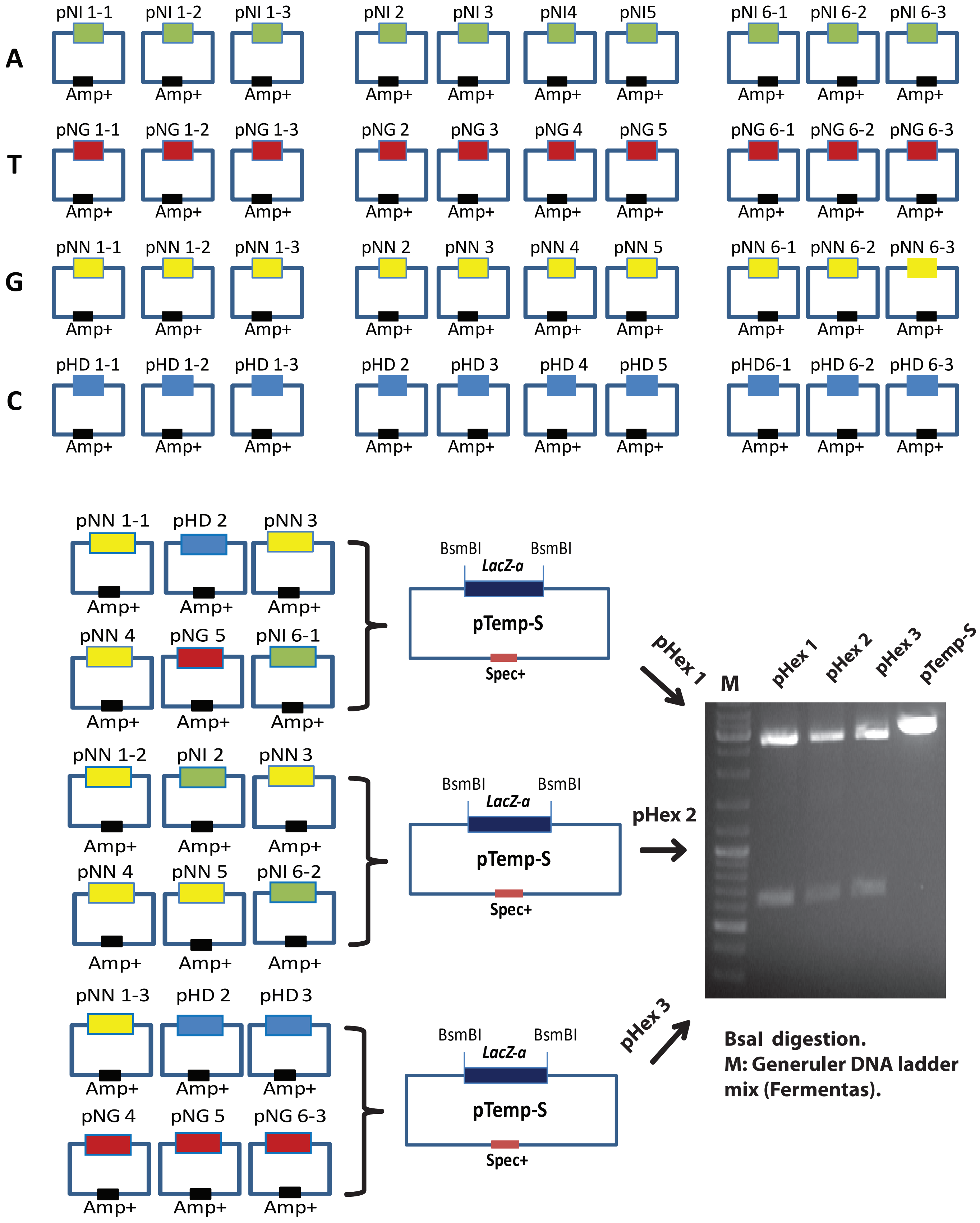

Figure S1. Repeat variable di-residue (RVD) plasmid library and hexamer assembly. Upper: RVD library plasmids for TALEN assembly. Lower: example of assembly of 3 hexamers for the left TALEN of exon 1 of Ddx3x.
