# Supplementary material for: Rapid Assembly of Customized TALENs into Multiple Delivery Systems
Source: PLoS One. 2013 Nov 7;8(11):e80281. doi: 10.1371/journal.pone.0080281 (PMC3820630; doi:10.1371/journal.pone.0080281)
Supplement: Figure S2 — TALENs’ target sequences of Ddx3x, Ddx3y and D1Pas1. The TALEN pair for Ddx3x targets the 5’ untranslated region. The TALEN pair for Ddx3y targets intron 4 (left TALEN, red) and exon 5 (Right TALEN, blue). The TALEN pair of the D1Pas1 (retrotransposed autosomal copy of Ddx3x) targets sequences corresponding to exon 1 (for left TALEN, red) and exon 2 (for right TALEN, blue) of Ddx3x. The left and right TALEN target sequences for each gene are indicated with bold underlines. The assembled RVDs (with encoded nucleotides) for the left and right TALENs are below each target gene region. RVDs (NI, NG, NN and HD) are color coded in correspondence with the RVD library in Figure S2. Exon sequences are indicated with bold font. (PDF) [file pone.0080281.s002.pdf]

Ddx3x Exon 1

5'... tgcggtagag gcagcctttg ctcagcagcg gaagactccg atttctcggt actcttca ...3'
3'... acgccarctc cgtcggaaac gagtcgtcgc cttctgaggc taaagagcca tgagaagt ...5'

	Hex-1	Hex-2	Hex-3
RVDs for left TALEN	g c g g t a<br>NNHDNNNNNGNI	g a g g c a<br>NN NINNNNHDNI	g c c t t t<br>NN HDHDNGNGNG

	Hex-4	Hex-5	Hex-6
RVDs for left TALEN	g a a g a g<br>NNNINININNNINN	t a c c g a<br>NGNIHDHDNNNI	g a a a t c<br>NNNINININGHD

Ddx3y Intron4/Exon 5

5'...tcttgattat tcgctgttc ag**GTTCGATG ATCATGGTCG AAATGACTAT GATGGTA** ...3'
3'... agaactaata agcgacaag tcCAAGCTAC TAGTACCAGC TTTACTGATA CTACCAT ...5'

	Hex-1	Hex-2	Hex-3
RVDs for left TALEN	c t t g a t<br>HDNGNGNNNING	t a t t c t<br>NGNINGNGHDNG	g c t g t t<br>NNHDNGNNNGNG

	Hex-4	Hex-5	Hex-6
RVDs for Right TALEN	A C C A T C<br>NIHDHDNINGHD	A T A G T C<br>NINGNINNNGHD	A T T T C G<br>NINGNGNGHDNN

D1Pas1

5'... **TGAGCTCGGG CTGGACCAGC AGTTGGCTGG CCTAGACCTG ACGTCTCGGG ACAGCCA** ...3'
3'... ACTCGAGCCC GACCTGGTCG TCAACCGACC GGATCTGGAC TGCAGAGCCC TGTCGGT ...5'

	Hex-1	Hex2	Hex3
RVDs for left TALEN	G A G C T C<br>NNNINIHDNGHD	G G G C T G<br>NNNNNNHDNGNN	G A C C A G<br>NNNIHDHDNINN

	Hex-4	Hex-5	Hex-6
RVDs for left TALEN	G G C T G T<br>NNNNHDNGNNNG	C C C G A G<br>HDHDHDNNNINN	A C G T C A<br>NIHDNNNGHDNI

Figure S2. TALENs' target sequences of Ddx3x Exon 1, Ddx3y and D1Pas1. The TALEN pair for Ddx3x targets the 5' untranslated region. The TALEN pair for Ddx3y targets intron 4 (left TALEN, red) and exon 5 (Right TALEN, blue). The TALEN pair of the D1Pas1 (retrotransposed autosomal copy of Ddx3x) targets sequences corresponding to exon 1 (for left TALEN, red) and exon 2 (for right TALEN, blue) of Ddx3x. The left and right TALEN target sequences for each gene are indicated with bold underlines. The assembled RVDs (with encoded nucleotides) for the left and right TALENs are below each target gene region. RVDs (NI, NG, NN and HD) are color coded in correspondence with the RVD library in Figure S1. Exon sequences are indicated with bold font.
