# Supplementary material for: Rapid Assembly of Customized TALENs into Multiple Delivery Systems
Source: PLoS One. 2013 Nov 7;8(11):e80281. doi: 10.1371/journal.pone.0080281 (PMC3820630; doi:10.1371/journal.pone.0080281)
Supplement: Protocol S1 — Protocol. (PDF) [file pone.0080281.s003.pdf]

Protocol S1.

A. Assembly of TAL effector RVDs into TALEN expression Entry vectors.

Step 1. Assembly of Hexamers.

1. Pick six TAL RVD plasmids, 200-250 ng for each, 200 ng pTemp-S.
2. Add 2 µl 10x T4 DNA ligase buffer, 1 ul BsmBI (New England Biolabs, R0580), and make up to 20 µl with ddH_2O.
3. Incubation 60 minutes at 55°C.
4. Add 1 µl T4 DNA ligase (New England Biolabs, M0202). Then run cycle as: 37° C 5min, 16 °C 10 min, total 9 cycles, followed by 16 °C 20 min .
5. Add Plasmid-Safe nuclease (Epicentre, E3101K) 1 µl into each reaction, then incubate 30-60 min at 37° C. Take out 6 µl of each ligation product and save at -20 °C. The rest of three hexamer ligation products for each side TALEN are combined into ONE tube.
6. Add 8 µl QIAEX II Suspension into each tube and purify through QIAEX II Gel Extraction Kit (Qiagen) following the manual.
7. Elute DNAs in 13 µl EB buffer (~11.5 µl may be recovered).

Step 2. Assembly of TALEN.

1. Add 100 ng (1 µl) TALEN expression Entry vectors which contains the last 0.5 appropriate RVD into the tube containing purified hexamer mixture, 1.5 10x T4 DNA ligase buffer, 1 µl BsaI, 1 µl T4 DNA ligase (New England Biolabs, #M202S), total volume 15 µl.
2. Run the second golden gate ligation at 37° C 5min, 16° C 10 min, total 9 cycles, followed by 37 °C 15 min, 80 °C 10 min.
3. Transform 7 µl ligation product into 50 µl chemically competent DH 5ɑ cells or other chemically competent cells.
4. Plate the cells on a Kan+ LB-agar plate.
5. Pick up the colonies to grow plasmids.
6. Digest the plasmid by using SalI.

B. **Example of restriction enzymes digestion patterns of different TALEN vectors.**

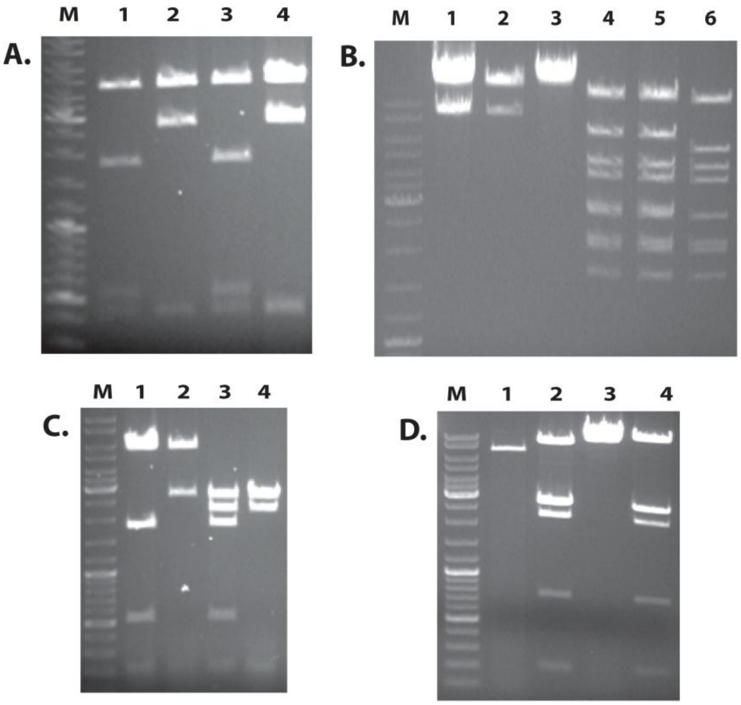

A. Entry plasmids. Lanes 1 and 3, pENTR-EF1a-TALEN-NN-T2A-EGFP and pENTR-EF1a-TALEN-NN-T2A-mCherry. Lanes 2 and 4, pENTR-EF1a-Ddx3x-TALEN (L)-T2A-EGFP and pENTR-EF1a-Ddx3x-TALEN (R)-T2A-mCherry. Restriction enzyme: Sal I.
B. Adenoviral plasmids. Lanes 1, 2, 4 and 5, pAd-EF1a-Ddx3x-TALEN (L)-T2A-EGFP and pAd-EF1a-Ddx3x (R)-TALEN-T2A-mCherry. Lanes 3 and 6, pAd/PL-DEST. Restriction enzymes: SnaB I for Lanes 1 to 3. PvuI for Lanes 4 to 6.
C. Entry plasmids for "2 in 1" system. Lanes 1 and 3, pL1R5-CAG-EGFP-T2A-TALEN-NN-BGHpA and pL5L2-EF1α-mCherry-T2A-TALEN-NN. Lanes 2 and 4, pL1R5-CAG-EGFP-Ddx3x-TALEN (L)-BGHpA and pL5L2-EF1a-mCherry-T2A-Ddx3x-TALEN (R). Restriction enzyme: SalI.
D. TALEN "2 in 1" plasmids. Lanes 1 and 3, Destination plasmids pPB-DEST and pPB-Puro-DEST. Lanes 2 and 4, pPB-Ddx3x-TALEN and pPB-Ddx3x-TALEN-Puro. Restriction enzyme: Sal I.

M: Generuler DNA ladder mix (Fermentas).
