# Supplementary material for: Rapid Assembly of Customized TALENs into Multiple Delivery Systems
Source: PLoS One. 2013 Nov 7;8(11):e80281. doi: 10.1371/journal.pone.0080281 (PMC3820630; doi:10.1371/journal.pone.0080281)
Supplement: Table S1 — Comparison of the Strategy of TALEN Assembly. (PDF) [file pone.0080281.s004.pdf]

Supplementary Table S1. Comparison of the Strategy of TALEN Assembly

	Plasmid number for RVD assembly (Includes RVD plasmids and intermediate plasmids, but not the TALEN backbones)	RVD assembly		TALEN expression vector	RVD number in TALEN (one side)	Transformation
		PCR Amplification and Purification	Plasmid module cloning			
Sanjana NE, et al [15].	4	Yes. Golden-gate ligation to form three hexamers , then amplified hexamers.		Golden-gate ligation.<br><br>CMV promoter for mammalian cells.	19.5	one
Uhde-Stone C, et al [54] .	4	Yes. Golden-gate ligation to form two hexamersn and one quatromer, then amplified hexamers and quatromer		Golden-gate ligation.<br><br>CMV, EF1a and MSCV promoter for mammalian cells.	17.5	one
Miller J, et al [20] .	Not clear	Yes. Amplified individual module, then formed TALE through overlap PCR.		Ligation.<br><br>CMV promoter for mammalian cells.	12.5	one
Li T, et al [24].	4	Yes. Golden-gate ligation to form octomers. Then the octomers were digested by different restriction enzymes.		Ligation	15.5 or 23.5	one

Zhang Z, et al [55].	256	Amplified two tetramers		Ligation<br><br>EF1a promoter for mammalian cells.	21.5	one
This work	41	No	Golden-gate ligation	Golden-gate ligation.<br>EF1a and CAG promoter for mammalian cells.<br>With reporter genes in vectors.<br>Can be transferred to other delivery system through Gateway recombination.	19.5	one
Weber E, et al [56].	71	No	Golden-gate ligation	Golden-gate ligation	17.5	two
Cermak T, et al [18].	68	No	Golden-gate ligation	Golden-gate ligation<br><br>CMV promoter for mammalian cells.	Variable	two
Schmid-Burgk J, et al [38]	67	No	Ligation-independent. Multiple steps of plasmid digestion and purification.	Ligation<br><br>CMV promoter for mammalian cells.	19.5	two
Kim Y, et al [36].	424	No	Golden-gate ligation<br><br>CMV promoter for mammalian cells.		14.5-18.5	one
Reyon D, et al [16].	376	Yes. Plus multiple steps of plasmids digestion, purification and ligation. Appropriate for large scale TALEN assembly through automation platform .		Ligation<br><br>CMV promoter for mammalian cells.	Variable	one
